# Superferromagnetic Disk Particles for Magnetic Particle Imaging

**DOI:** 10.1002/smtd.202501349

**Published:** 2025-11-23

**Authors:** Erik M. Mayr, Justin Ackers, Alexander Gogos, Subas Scheibler, Michal Krupinski, Matthias Graeser, Inge K. Herrmann, Hans J. Hug

**Affiliations:** ^1^ Nanoparticle Systems Engineering Laboratory Department of Mechanical and Process Engineering ETH Zurich Sonneggstrasse 3 8092 Zurich Switzerland; ^2^ Faculty of Medicine University of Zurich Raemistrasse 71 8006 Zurich Switzerland; ^3^ Ingenuity Lab Balgrist University Hospital and University of Zurich Forchstrasse 340 8008 Zürich Switzerland; ^4^ Nanomaterials in Health Laboratory Swiss Federal Laboratories for Materials Science and Technology (Empa) Lerchenfeldstrasse 5 9014 St. Gallen Switzerland; ^5^ Magnetic & Functional Thin Films Laboratory Swiss Federal Laboratories for Materials Science and Technology Empa Ueberlandstrasse 129 8600 Dübendorf Switzerland; ^6^ Fraunhofer IMTE Fraunhofer Research Institution for Individualized and Cell‐Based Medical Engineering Mönkhofer Weg 239a 23562 Lübeck Germany; ^7^ Institute of Nuclear Physics Polish Academy of Sciences Radzikowskiego 152 31‐342 Kraków Poland; ^8^ Chair of Metrology University Rostock Albert‐Einstein‐Str. 2 18059 Rostock Germany; ^9^ Department of Physics University of Basel Klingelbergstrasse 82 4056 Basel Switzerland

**Keywords:** magnetic disk particles, magnetic nanoparticles, magnetic particle imaging, super ferromagnetism

## Abstract

Magnetic particle imaging (MPI) offers rapid, highly sensitive tracer‐based imaging without ionizing radiation, but its clinical translation remains challenged by the limited performance of existing superparamagnetic (SP) tracers. In this work, precisely engineered disk‐shaped nanoparticles fabricated from superferromagnetic (SF) discontinuous metal–insulator multilayers (DMIMs) consisting of SP nanoscale metal islands embedded in a nonmagnetic oxidic matrix are introduced. By leveraging inter‐island exchange interactions, robust SF behavior is achieved in extended DMIMs, exhibiting exceptionally high susceptibilities and a sharp magnetization switching at fields below 1 mT. When evaluated in MPI experiments, magnetic disk particles (MDP) with a diameter of 1800 nm patterned from the SF DMIMs demonstrate up to a 1.6‐fold improvement in spatial resolution and a 2.4‐fold increase in sensitivity compared to Perimag tracers, the current gold standard in MPI. System matrix measurements and hybrid MPI reconstructions further highlight the superior imaging characteristics of MDP tracers in complex, clinically relevant geometries. These findings establish SF DMIM disks as a promising next‐generation tracer platform for MPI, with the potential to simplify scanner designs and to pave the way for a clinical translation of MPI.

## Graphical abstract

Superferromagnetic disk particles (MDPs), fabricated by a top‐down approach from discontinuous metal–insulator multilayers, represent an entirely new tracer concept for MPI. In this first demonstration, MDPs already surpass the current gold‐standard Perimag tracers in sensitivity and spatial resolution. Importantly, the underlying design principles reveal a clear pathway to further reduce particle size and drastically enhance performance, opening the door to next‐generation, clinically viable MPI contrast agents.

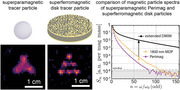

## 1 Introduction

**Figure 1:**
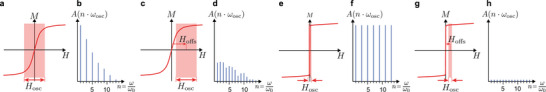
Signal Generation in MPI: a) An applied oscillatory magnetic field *H*_osc_ with frequency ω_osc_ drives a localized distribution of superparamagnetic particles through their non‐linear, ideally hysteresis‐free Langevin‐type *M*(*H*)‐loop (red line). The applied field amplitude (pale red shaded area) must be sufficiently large to drive the particles towards the saturated parts of the *M*(*H*)‐curve. b) The non‐linear *M*(*H*)‐behavior generates a series of odd higher harmonic induction signals, which are induced in one or more pick‐up coils, typically used for tracer detection. The amplitudes *A*((2*n* + 1) · ω_osc_) decay rapidly with increasing harmonic number *n*. c) Spatial resolution is achieved by applying a magnetic field gradient such that mainly particles located in the field‐free region (*H*_offs_ = 0) contribute to the higher harmonic response (b), while particles at a finite offset field *H*_offs_ > 0 produce reduced harmonic signals (d). Notably, even harmonics also appear for *H*_offs_ ≠ 0. e) Particles with a steeper, more step‐like *M*(*H*)‐response allow for reduced oscillatory field amplitudes *H*_osc_, while still efficiently probing the non‐linear regime. f) A steeper *M*(*H*) response also leads to a slower decay of the higher harmonic amplitudes *A*((2*n* + 1) · ω_osc_) with increasing harmonic number *n*. g) A smaller offset field is sufficient to saturate tracers with a steeper *M*(*H*)‐response. h) Consequentially, at nonzero offset fields *H*_offs_ ≠ 0 the generation of higher harmonic signals is strongly attenuated.

Magnetic Particle Imaging (MPI) is a non‐ionizing, background free imaging modality that offers high sensitivity and rapid imaging capabilities.^[^ ^1^, ^2^, ^3^, ^4^ ^]^ These advantages make MPI a promising tool for a variety of clinical applications, including the guidance of cardiovascular interventions,^[^ ^5^ ^]^ stroke monitoring,^[^ ^6^ ^]^ and functional neuroimaging.^[^ ^7^ ^]^ MPI leverages the non‐linear magnetization properties of magnetic nanoparticles, which serve as tracers. A time‐varying (typically sinusoidal) excitation field periodically drives the tracers' magnetization towards and away from saturation (**Figure** **1** **a**). This non‐linear response leads to the generation of higher harmonic components in the resulting signal, typically an induction signal in pickup coils (Figure 1b). Biological tissues, being paramagnetic or diamagnetic, do not produce such higher harmonics, rendering MPI inherently background‐free. To obtain spatial resolution, a magnetic gradient field is applied to create a field‐free region (FFR), either a point or a line, within the imaging volume. Only tracer particles located within this region, where the local magnetic offset field is approximately zero, fully respond to the excitation field and generate strong higher harmonic signals. In contrast, tracers located outside the FFR experience a static offset field (indicated by the dashed red arrow in Figure 1c), become partially saturated and produce weaker higher harmonic signals (Figure 1d). Notably, the spectral content of the signal reflects this offset: for *H*_offs._ ≠ 0, both even and odd harmonics are present, whereas in the FFR, only odd harmonics appear in the particle spectrum. The amplitudes and phases of these harmonics are influenced by the applied field gradients and encode the spatial distribution and characteristics of the tracer particles, enabling high‐resolution, real‐time 3D imaging.^[^ ^1^, ^3^ ^]^

In MPI, spatial resolution is primarily governed by the strength of the applied magnetic field gradient and the magnetic susceptibility $\chi ={\left.\frac{dM}{dH}\right|}_{H=0}$ of the tracers.^[^ ^3^ ^]^ Tracers with a steep *M*(*H*)‐response (i.e., high χ) produce stronger higher harmonic signals (Figure 1e,f), thereby enhancing both sensitivity and spatial resolution for a given gradient strength. For a set target resolution, such tracers permit the use of weaker gradients and lower drive field amplitudes (Figure 1g,h), simplifying hardware requirements and reducing potential risks for patients.

Typically, superparamagnetic iron oxide nanoparticles (SPIONs) are used as tracers.^[^ ^8^, ^9^, ^10^ ^]^ These SPIONs exhibit a Langevin‐type *M*(*H*)‐response (red line in Figure 1a,c) with a limited susceptibility and a modest saturation magnetization *M*_s_ < 480 kAm^−1^.^[^ ^11^ ^]^ The resulting suboptimal imaging performance of currently available tracers necessitates substantial magnetic gradient fields of 2–7 T m^−1^ to achieve resolutions in the mm‐range in MPI scanners for small animal imaging.^[^ ^4^, ^8^, ^12^ ^]^ Generating such strong gradients over larger imaging volumes needed for human imaging poses significant challenges, including immense electrical power consumption requiring substantial cooling,^[^ ^13^ ^]^ mechanically complex permanent magnet arrangements,^[^ ^14^ ^]^ or superconductor‐based solutions.^[^ ^15^ ^]^ These technical challenges have impeded the adoption of MPI as a routine imaging tool in human medicine.^[^ ^16^ ^]^ Therefore, developing tracer particles with a significantly sharper *M*(*H*) response is essential for advancing MPI toward medical applications, either making it possible to reach the current imaging resolution with significantly simpler scanners or boosting the resolution at the same gradient strengths.

A promising approach to overcoming the limitations of superparamagnetic tracers was recently demonstrated by Tay et al. ^[^ ^17^ ^]^ In their study, several SPIONs were encapsulated into micron sized micelles and arranged into linear chains using a pre‐polarization pulse. Magnetostatic inter‐particle interactions within the chains stabilized the magnetization of individual particles along the chain direction, inducing a collective superferromagnetic (SF) state characterized by sharp magnetization switching. This sharper switching at a coercive field of μ_0_ *H*_c_ ≈ 13 mT resulted in an 40‐fold increased sensitivity and about 10‐fold spatial resolution in MPI experiments compared to the same SPIONs in their superparamagnetic state. Although these results highlight the promising potential of SF nanoparticle assemblies as MPI tracers, their signal generation critically depends on the application of pre‐polarizing field pulses and on excitation fields of sufficiently large amplitude to induce magnetization reversal. Beyond the associated hardware demands, sinusoidal drive fields exceeding 10mT at typical MPI frequencies may induce undesired physiological responses such as peripheral nerve stimulation (PNS).^[^ ^18^, ^19^, ^20^ ^]^ Furthermore, efficient tracer performance requires alignment of the drive field with the chain axis, as SF magnetization reversal only occurs along this direction. Excitation applied orthogonally leaves the chains magnetically inactive and may even compromise their structural integrity. This pronounced directional dependence renders such tracer systems incompatible with MPI implementations that employ multidimensional excitation fields for rapid volumetric imaging.^[^ ^2^, ^21^ ^]^

An ideal next‐generation tracer would exhibit SF behavior with minimal dynamic coercivity and a strong, direction‐independent signal response. In this work, we present precisely designed disk‐shaped particles comprised of discontinuous metal–insulator multilayers (DMIMs) that exhibit the desired SF characteristics in a mechanically rigid format, eliminating the need for prepolarization pulses. Our results demonstrate that these SF disk particles significantly outperform Perimag, a widely used benchmark tracer, in both spatial resolution and sensitivity. By enhancing MPI performance by orders of magnitude, SF DMIM disks could enable the development of next‐generation medical MPI scanners with dramatically improved imaging capabilities and broader clinical applicability.

## 2 Results and Discussion

As discussed by Bedanta et al.,^[^ ^23^ ^]^ superferromagnetic (SF) states can emerge in ensembles of superparamagnetic (SP) nanoparticles when inter‐particle interactions are present. In linear arrangements of SP particles, such as chains,^[^ ^17^ ^]^ SF behavior can arise from dipolar coupling via their mutual stray fields. In two‐ and three‐dimensional arrangements; however, stray field interactions alone are insufficient to induce SF order; instead, direct magnetic exchange interactions between neighboring SP particles are necessary to stabilize the SF state. This requirement can be met in discontinuous metal–insulator multilayers (DMIMs),^[^ ^23^ ^]^ which consist of SP nanoscale metal islands embedded in a non‐magnetic matrix. By precisely tuning the individual nominal layer thicknesses, it is possible to induce the inter‐island exchange coupling necessary for SF behavior. Specifically, achieving a low‐coercivity, sharply switching *M*(*H*)‐loop requires sufficiently strong inter‐island exchange interactions within a CoFe layer or between adjacent CoFe layers. The onset of intra‐layer exchange occurs when the CoFe layer exceeds a critical nominal thickness, leading to collective SF behavior. Alternatively, when the CoFe thickness is kept below this threshold, SF order can still be realized by reducing the Al_2_O_3_ spacer thickness to promote inter‐layer exchange. In both cases, careful adjustment of the metal and insulator thicknesses determines whether the system exhibits superparamagnetic or superferromagnetic properties, which directly impacts tracer performance in MPI.

Building on this concept and leveraging the precise control over layer thicknesses and the flexibility in material selection afforded by physical vapor deposition techniques, we employ magnetron sputtering to fabricate extended DMIM films with tailored magnetic architectures exhibiting SF properties. Layer thicknesses reported throughout this work are given as nominal values. These correspond to the thickness of a hypothetical, defect‐free, single‐crystalline layer of the respective material with its tabulated bulk density. In discontinuous CoFe layers, the granular microstructure leads to effective vertical grain dimensions in TEM cross‐sections that are typically larger than the nominal thickness. As the layers are not densely packed, thickness estimates based on TEM images are therefore unreliable and not suited for deposition control. Reporting nominal thickness values provides a consistent and reproducible basis for defining and tuning the DMIM architecture. Using polystyrene bead lithography, these DMIMs are subsequently patterned into magnetic disk particles (MDP). This micro‐patterning technique enables the top‐down fabrication of disk particles with diameters ranging from approximately 20 nm to several microns with high reproducibility and precise size control.^[^ ^24^, ^25^ ^]^ The MDPs are then released from the substrate and investigated as tracer candidates for high‐performance MPI.

### 2.1 Achieving Superferromagnetic Properties

**Figure 2:**
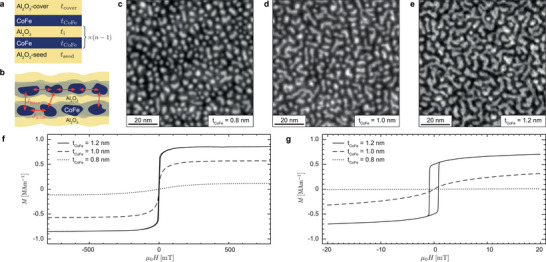
Magnetic Properties of discontinuous CoFe/Al_2_O_3_ multilayers: a) Schematic of the deposited discontinuous metal‐insulator multilayers (DMIMs) with nominal thicknesses *t*_seed_, *t*_CoFe_, *t*_i_, and *t*_cover_. b) Superferromagnetic properties can be realized by sufficiently strong intra‐layer (*J*_intra_) or inter‐layer (*J*_inter_) exchange coupling between neighboring nanoscale islands. This schematic was inspired by Figure 1 from reference [22]. c–e) Planar *Z*‐contrast scanning transmission electron microscopy micrographs of Ti(5)/Al_2_O_3_(3)/CoFe(*t*_CoFe_)/Al_2_O_3_(3) DMIMs (thicknesses in brackets in nanometers), sputter‐deposited onto a Si_3_N_4_ membrane window with *t*_CoFe_ = 0.8, 1.0 , and 1.2 nm. f,g) Room‐temperature *M*(*H*)‐curves measured through vibrating sample magnetometry for DMIMs with *t*_CoFe_ = 0.8 (dotted), 1.0 (dashed), and 1.2 nm (solid line). In f), the curves are measured over a wide field range of ±800 mT, showing Langevin‐like behavior for the superparamagnetic DMIMs with *t*_CoFe_ = 0.8 and 1.0 nm and the onset of hysteretic behavior for *t*_CoFe_ = 1.2 nm attributed to a superferromagnetic coupling between islands. However, the small SF hysteresis is not visible at this field scale. Therefore, g) shows the corresponding *M*(*H*)‐loops measured over a reduced field range of ±20 mT, highlighting the transition from SP to SF behavior, with the emergence of a clear hysteresis for the *t*_CoFe_ = 1.2 nm DMIM.

To obtain superferromagnetic (SF) behavior, we fabricated discontinuous metal–insulator multilayers (DMIMs) with the structure ${\mathrm{Al}}_{2}O_{3}\left(t_{\mathrm{seed}}\right)/$ ${\left[\mathrm{CoFe}\left(t_{\mathrm{CoFe}}\right)/{\mathrm{Al}}_{2}O_{3}\left(t_{i}\right)\right]}_{\times (n-1)}$/CoFe (*t*_CoFe_)/${\mathrm{Al}}_{2}O_{3}\left(t_{\mathrm{cap}}\right)$, as schematically shown in **Figure** **2** **a**. For optimal magnetic performance, we used Co_40_Fe_60_, a cobalt–iron alloy that exhibits the highest known saturation magnetization of $M_{s}^{\mathrm{bulk}}=$ 1.95MAm^−1^, approximately five times greater than that of iron oxides.^[^ ^11^ ^]^ The high surface energy of Al_2_O_3_ promotes the formation of discontinuous metal layers, leading to isolated superparamagnetic islands at lower CoFe thicknesses (*t*_CoFe_).^[^ ^22^, ^26^ ^]^ In such a DMIM system intra‐layer and inter‐layer exchange coupling (*J*_intra_ and *J*_inter_) can be present (Figure 2b). Increasing *t*_CoFe_ enhances *J*_intra_, while reducing the insulator thickness *t*_i_ promotes *J*_inter_.

To assess the morphology of the metal islands, we performed scanning transmission electron microscopy (STEM) on simplified trilayers Ti(5)/Al_2_O_3_(3)/CoFe(*t*_CoFe_)/Al_2_O_3_(3) deposited on Si_3_N_4_ windows (thicknesses in brackets are given in nm). The obtained images reveal well‐separated CoFe islands for *t*_CoFe_ = 0.8 and 1.0 nm (Figure 2c,d), while thicker layers (*t*_CoFe_ = 1.2 nm) lead to larger, coalescing grains (Figure 2e).

Figure 2f shows room temperature vibrating sample magnetometry (VSM) measurements of [Al_2_O_3_(3)/CoFe(*t*_CoFe_)]_× 4_/Al_2_O_3_(3) DMIMs acquired over a field range of ±800 mT. The measurements highlight the transition from SP behavior at lower CoFe thicknesses (*t*_CoFe_ = 0.8 and 1.0 nm; dotted and dashed curves) to SF behavior at *t*_CoFe_ = 1.2 nm (solid curve). Additionally, a pronounced increase of the saturation magnetization *M*_s_ from 0.28 to 0.85 MAm^−1^ is observed as *t*_CoFe_ is increased from 0.8 to 1.2 nm, which we attribute to the presence of dead magnetic layers at the metal/oxide interfaces.^[^ ^27^, ^28^ ^]^ The dead magnetic layer thickness is determined to be $t_{\mathrm{dml}}=\left(0.56\pm 0.04\right)\;\mathrm{nm}$ (see Supporting Information).

VSM measurements acquired with a reduced field range of ±20 mT (Figure 2g) provide further details on the SF properties observed for *t*_CoFe_ = 1.2 nm. The corresponding *M*(*H*)‐loop (solid curve) reveals a hysteretic magnetization response with a sharp magnetization switching at a coercive field of μ_0_ *H*_c_ ≈ 0.85 mT. At fields beyond the coercive field (|μ_0_ *H*| > *H*_c_), the magnetization continues to increase gradually from 0.53 to 0.85 MAm^−1^ as the field is raised to approximately 300 mT, indicating a residual SP contribution. This behavior is attributed to the coexistence of SF and SP regions within the CoFe layers, originating from exchange‐coupled islands and isolated, non‐coupled islands, respectively. This SP background can be suppressed by increasing magnetic exchange interactions, albeit at the cost of broader hysteresis. This can be achieved either by increasing the nominal CoFe thickness (enhancing intra‐layer exchange *J*_intra_) or by reducing the Al_2_O_3_ spacer thickness (enhancing inter‐layer exchange *J*_inter_).

The multilayer structure of the DMIMs employed for particle fabrication is given by Al_2_O_3_(3)/[CoFe(1)/Al_2_O_3_(0.75)]_×9_/CoFe(1)/Al_2_O_3_(3). In these DMIMs, SF properties emerge through intra‐layer exchange coupling (*J*_intra_) across the 0.75 nm Al_2_O_3_ spacers, as for a CoFe layer with a nominal thickness of 1 nm, inter‐layer exchange coupling alone does not establish a SF state (Figure 2g).

### 2.2 Nanoparticle Fabrication and Characterization

**Figure 3:**
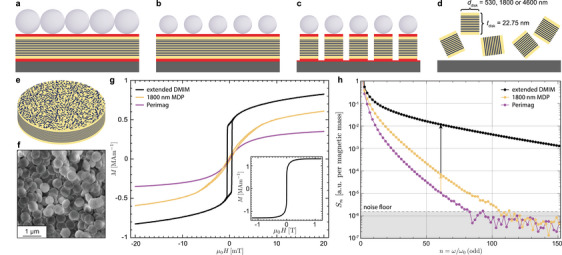
Fabrication and characterization of SF magnetic disk nanoparticles: a) Schematic of the patterning process: polystyrene (PS) beads with respective initial diameters of 5310, 2230, and 658 nm are self‐assembled into a close‐packed hexagonal array atop the DMIMs. These are supplemented with sacrificial Ge layers. b) The PS bead diameters are reduced by oxygen plasma etching to define the final disk diameters of 4600, 1800, and 530 nm, respectively. c) PS beads act as an etch mask during ion‐beam etching, transferring the pattern into the DMIM and partially into the bottommost Ge layer, thereby forming multilayered magnetic disks beneath the beads. d) The disk particles are released from the substrate by dissolving the sacrificial Ge layers in a 35% H_2_O_2_ solution. e) Schematic of a resulting DMIM disk particle, illustrating its multilayer structure with discontinuous magnetic layers. f) SEM image of DMIM disk particles re‐deposited onto a wafer from suspension. g) *M*(*H*)‐loops measured over a field range of ±20 mT for the extended DMIMs (black), DMIMs patterned into SF disk particles (yellow) and Perimag in suspension (purple) using a vibrating sample magnetometer. The magnetization was calculated by dividing the measured magnetic moment by the volume of the magnetic material. h) Odd higher harmonic amplitudes, measured by magnetic particle spectroscopy (excitation frequency: 25 kHz, amplitude: μ_0_ *H* = 20 mT) for the extended DMIMs (black) as well as suspension of DMIM MDPs (yellow) and Perimag (purple), normalized to the magnetic mass.

Disk‐shaped nanoparticles were fabricated by self‐assembling commercially available polystyrene (PS) spheres onto the DMIMs (**Figure** **3**a).^[^ ^29^ ^]^ The PS spheres formed a close‐packed hexagonal array with minimal defects across the 2‐inch wafer. Their diameters were then reduced to the desired diameters by oxygen plasma etching (Figure 3b). Subsequent, the beads were used as a mask in the ion beam etching to pattern the DMIMs into nanoscale disks (Figure 3c). To release the disk particles, the 50 and 20 nm thick bottom‐ and top‐sacrificial Ge layers were dissolved in a H_2_O_2_ solution (Figure 3d).

A schematic of the fabricated disk‐shaped particles and a scanning electron microscopy (SEM) image of disks with a diameter of 530 nm are shown in Figure 3e,f. In this study, we focused on the magnetic properties and imaging performance of 1800 nm disks, which offer a favorable combination of signal strength and particle miniaturization. Additional data on particles with smaller and larger diameters, including their MPI performance, are provided in Supporting Information.

We first compared the magnetization characteristics of the selected 1800 nm MDPs with those of a commercial MPI tracer, Perimag. The Langevin‐type *M*(*H*)‐response of Perimag (purple curve in Figure 3g), measured over a ±20mT field range, shows a gradual increase to *M* = 0.36 MAm^−1^ and a susceptibility of approximately $\chi_{\mathrm{VSM}}^{\mathrm{Perimag}}\approx 123$. In comparison, the 1800 nm MDPs (yellow curve) exhibit both a higher magnetization and an increased susceptibility, $\chi_{\mathrm{VSM}}^{\mathrm{MDP}}\approx 139$. Accordingly, the magnetic particle spectra recorded for the MDPs (drive frequency: 25 kHz, peak field amplitude: μ_0_ *H* = 20 mT)^[^ ^30^ ^]^ showed significantly enhanced higher harmonic signal amplitudes compared to Perimag (yellow and purple data in Figure 3h). With a slower decay of high‐frequency components, the MDPs generate signal amplitudes almost one order of magnitude larger than Perimag for the 61^st^ harmonic (yellow arrow in Figure 3h), highlighting the great potential of superferromagnetic disk particles for MPI applications.

Although the MDPs exhibit both a higher saturation magnetization and improved susceptibility compared to Perimag, they do not retain the narrow, sharply switching hysteresis loop observed in the extended DMIMs that feature a large susceptibility of $\chi_{\mathrm{DMIM}}^{\mathrm{VSM}}\approx 18\;300$ at a coercive field of μ_0_ *H*_c_ = 0.5mT (black curve in Figure 3g). We attribute this to shape‐dependent demagnetization effects arising from the disk geometry, as discussed in detail in Supporting Information. The inset shows the *M*(*H*)‐loop of the extended DMIM over a wider field range of ±1.3 T, again revealing the coexistence of hysteretic SF and SP phases in DMIMs. Importantly, the potential for further signal enhancement is underscored by the even stronger MPS response measured for the extended DMIM structure (black data points in Figure 3h). Specifically, at the 61st harmonic, the spectrum of the extended DMIM exceeds that of Perimag by more than three orders of magnitude (black arrow in Figure 3h). Further, while the Perimag spectrum reaches the noise floor at approximately the 80th harmonic, the extended DMIM remains about three orders of magnitude above the noise floor even at the 151st harmonic, corresponding to a frequency of 3.78 MHz.

These results highlight a clear pathway for optimizing tracer performance, suggesting that future work should focus on developing superferromagnetic disk particles with ultrathin magnetic layers to mitigate demagnetization effects and fully exploit the potential of superferromagnetic DMIM disk particles as MPI tracers.

### 2.3 Advancements in Magnetic Particle Imaging Performance

**Figure 4:**
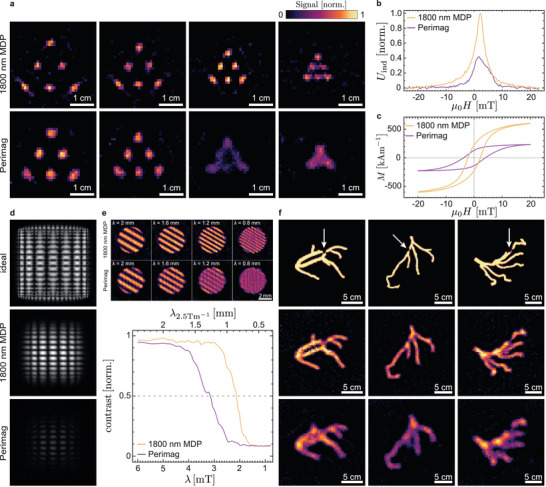
MPI, MPS, and hybrid MPI characterization: a) MPI reconstructions of triangular well arrays with center‐to‐center spacings of *d* = 10, 8, 6, and 4 mm, filled with either MDP (top row) or Perimag (bottom row) tracer suspensions. The color scale is individually normalized for each subfigure for improved visibility. b) Point‐spread functions (PSFs) extracted from one‐dimensional MPS measurements for the MDPs (yellow) and Perimag (purple) tracers. c) Dynamic *M*(*H*) loops of the MDP and Perimag tracers, reconstructed from the one‐dimensional MPS data. d) Amplitude pattern of a representative frequency component (667.7 kHz, mixing order 27) of the system matrix. The top panel is calculated for an ideal tracer, center and bottom panels are measured for the MDPs and Perimag, using a two‐dimensional Lissajous excitation, shown at identical color scales for direct comparison. e) Top: Representative hybrid MPI reconstructions of sinusoidal tracer density distributions, described by Equation (1) with θ = 60°. Bottom: Modulation transfer functions derived from hybrid MPI reconstructions at different spatial wavelengths. f) Top: Software phantom images of coronary artery projections derived from CT data, with artificially introduced stenoses indicated by white arrows. Center and bottom: corresponding hybrid MPI reconstructions obtained using the system matrices of the MDP and Perimag tracers, respectively.

To assess the imaging performance of the 1800 nm‐diameter MDPs, various MPI and MPS techniques were employed. Since the quantitative characterization of the MPI performance of a tracer across different measurement techniques and instruments remains challenging,^[^ ^10^ ^]^ we directly compared our SF DMIM disk particles with Perimag, a widely used SP tracer that serves as the current gold standard in MPI. To ensure a fair comparison of MPI performance between SF and SP tracers, suspensions of MDPs and Perimag were prepared at identical magnetic mass concentrations of $c_{\mathrm{CoFe}}^{\mathrm{MDP}}=c_{\mathrm{Fe}}^{\mathrm{Perimag}}$ = 484 μg ml^−1^. A Bruker preclinical MPI system was then used to image 3D‐printed test samples consisting of circular wells with a diameter of 1.85 mm, arranged in triangular patterns with center‐to‐center spacings of 10, 8, 6, and 4  mm, filled with either the MDP or Perimag suspensions (**Figure** **4**a). Image reconstructions were performed in frequency space using measured two‐dimensional higher‐harmonic amplitude and phase data in the form of tracer specific system matrix calibrations.^[^ ^31^ ^]^ For details regarding the system matrix calibration and regularization procedures refer to Experimental Section and Supporting Information. At well spacings of 10 and 8 mm, the quality of the MPI images obtained with MDPs and Perimag is comparable. However, at reduced spacings of 6 and 4 mm, a clear improvement in spatial resolution is observed for the MDPs: the individual wells are distinctly resolved, while significant overlap occurs for Perimag.

A more quantitative assessment of spatial resolution and sensitivity in the case of one‐dimensional excitation at 25 kHz was conducted using the magnetic particle spectrometer, previously used for the measurements shown in Figure 3h. For the present experiments, the same setup was operated with an additional static offset field, enabling the direct measurement of point spread functions (PSFs). The resulting PSFs for the MDP and Perimag suspensions are shown as yellow and purple traces, respectively, in Figure 4b. The sensitivity and spatial resolution provided by a tracer particle are related to the peak signal intensity and full‐width at half‐maximum (FWHM) of the PSF, respectively.^[^ ^10^, ^32^ ^]^ Full‐width at half‐maximum (FWHM) values of 4.06 and 6.59 mT were obtained for the MDP and Perimag suspensions, respectively. This reflects an ≈1.6‐fold improvement in spatial resolution, along with a signal amplitude, and thus a sensitivity enhancement of about a factor of 2.4. These improvements already represent a substantial advance for a first demonstration of this new tracer concept. Remaining limitations can be attributed to demagnetization effects inherent to the disk geometry, as analyzed in the Conclusions and Perspectives section and further discussed in the Supporting Information.

Beyond these resolution metrics, the one‐dimensional MPS data also enables the reconstruction of the dynamic *M*(*H*) loops of the MDP and Perimag tracers, shown in Figure 4c (yellow and purple curves, respectively). The magnetization obtained at 20 mT for the MDPs agrees well with that extracted from VSM measurements shown previously in Figure 3g (yellow line). However, the dynamic *M*(*H*)‐loop exhibits a broader hysteresis compared to the quasi‐static VSM measurement. This broadening is attributed to the general increase in magnetic hysteresis at shorter measurement time scales, as thermal activation becomes less effective in assisting magnetization reversal. This likewise leads to a hysteretic magnetization response, as well as an additional reduction in the magnetization of Perimag (purple), as larger SP nanoparticles struggle to follow the rapidly oscillating external field. Nevertheless, the improvement of the susceptibilities observed here approximately corresponds to that of the VSM loops (Figure 3g) at low measurement frequencies.

A further evaluation of the improved imaging performance of the MDP tracer particles under two‐dimensional excitation was conducted using hybrid MPI techniques.^[^ ^33^ ^]^ This approach enables the simulation of MPI responses for complex tracer distribution geometries that are difficult to realize experimentally, for example, structures that are challenging to fabricate, hard to homogeneously fill with tracers, or that would currently require impractically large quantities of MDPs. The modeling is based on hybrid system matrices that are experimentally acquired using a magnetic particle spectrometer. These matrices offer higher resolution (i.e., greater pixel density) and an improved signal‐to‐noise ratio compared to those obtained directly from an MPI scanner. In this study, the hybrid SM were measured in a custom‐built multidimensional MPS system^[^ ^4^, ^34^ ^]^ employing a two‐dimensional Lissajous excitation field (drive frequencies: ≈25 kHz, peak field amplitudes: 12 mT), as well as offset fields covering a field of view (FOV) of 30mT/$\mu_{0}$. Further details are given in the Experimental Section.

A first insight in the achievable resolution can be gained via the density of the pattern structures of the system matrix components. The top panel of Figure 4d shows a Chebyshev polynomial‐like system matrix component (27^th^ harmonic, *f* = 19*f*_*x*_ + 8*f*_*y*_ ≈ 667.7 kHz) calculated for an ideal MPI tracer with a perfectly step‐like *M*(*H*)‐loop.^[^ ^3^ ^]^ The middle and bottom panels of Figure 4d show the system matrices obtained for MDP and Perimag tracers, respectively, for the same exemplary SM component in the same, normalized intensity‐scale. The comparison with the measured system matrices reveals that the system matrix measured for MDP tracers shows a much higher intensity and slower roll‐off toward the boundaries of the FOV than that obtained for Perimag. (For plots with individually normalized intensity scales and phase information, see Supporting Information.) The observed behavior is consistent with the behavior observed in the one‐dimensional MPS measurements shown in Figure 3f,h (compare yellow and purple curves).

For an additional resolution quantification, software phantoms of sinusoidal tracer density distributions with spatial wavelengths λ ranging from 2.4 mm down to 0.32 mm were simulated using the hybrid system matrices. The resulting hybrid MPI images for selected wavelengths are shown in Figure 4f,e (top part). The corresponding modulation transfer function (MTF) for the MDP and Perimag tracers is plotted in Figure 4e (bottom) as a function of spatial wavelength expressed in mT and, assuming a 2.5 T m^−1^ gradient, in mm (yellow and purple lines, respectively). Here, the spatial resolutions is defined as the respective wavelength at which the MTF retains half of its maximum contrast. Values of 0.86 and 1.29 mm are extracted for the MDP and Perimag tracers, respectively, corresponding to a 1.5‐fold improvement.

In order to evaluate the images obtained from the two tracers in clinically more relevant geometries, hybrid MPI imaging was performed on two‐dimensional coronary artery phantom samples derived computed tomography (CT) data, each modified to include an artificially introduced stenosis. The top row of Figure 4f presents the ground truth structures with the stenosis highlighted by the white arrows, while the middle and bottom rows display the reconstructed images based on the system matrices of the MDP and Perimag tracers, respectively. The reconstructions obtained with the MDP tracers reveal finer structural details, allowing clearer visualization of the coronary vessels and the introduced stenoses. In comparison, the reconstructions with Perimag tracers exhibit blurred vessel structures and reduced contrast, making the stenoses less distinguishable. These results demonstrate that MDP tracers can significantly enhance the diagnostic potential of MPI, as shown here through the successful imaging of fine vascular structures and simulated pathologies. Alternatively, their superior performance could be leveraged to maintain current image quality while reducing the required magnetic field gradients.

## 3 Conclusions and Perspectives

This study establishes MDPs, derived from superferromagnetic DMIMs, as a promising next‐generation tracer for MPI. By harnessing strong inter‐island exchange interactions within precisely engineered multilayers, the fabricated MDPs exhibit markedly superior sensitivity and spatial resolution compared to the current gold‐standard tracer, Perimag. Notably, 1800 nm‐diameter MDPs achieve up to a 2.4‐fold higher sensitivity and a 1.6‐fold improvement in spatial resolution compared to Perimag. This improvement remains below that obtained with superferromagnetic SPION chains embedded in vesicles as reported by Tay et al.^[^ ^17^ ^]^ However, unlike such assemblies, the superferromagnetic properties of our disk particles are intrinsic, do not require pre‐polarization pulses, and remain stable under arbitrary field orientations. Moreover, the dynamic coercivity of our SF MDPs is significantly lower—about five times less—than that reported by Tay et al., enabling their use with lower‐amplitude oscillating fields.

The comparison of extended films with MDPs of different diameters (4800, 1800, and 500 nm) further revealed that the observed orders‐of‐magnitude performance decay is caused by demagnetization effects, which scale with the particle's thickness‐to‐diameter ratio. This provides a clear strategy for improvement. Two complementary design routes can be distinguished: (i) the approach used in this work, where each CoFe layer is kept slightly below the intra‐layer superferromagnetic threshold and the Al_2_O_3_ spacer is reduced to promote inter‐layer exchange, resulting in a single, magnetically thick entity with slightly higher saturation magnetization; and (ii) an alternative route where the CoFe layers are made slightly thicker to achieve superferromagnetism within each individual layer, while employing sufficiently thick Al_2_O_3_ spacers to suppress inter‐layer coupling. Although the latter approach would slightly reduce the saturation magnetization, it would also diminish demagnetization fields and thus enhance the effective susceptibility. Based on the model in Equations (S1) and (S2) of the Supporting Information, susceptibilities of ≈1241 (for 1800 nm MDPs) and 363 (for 500 nm MDPs) are predicted for *t*_CoFe_ ≈ 1.2 nm. This corresponds to expected improvements in MPI sensitivity and resolution by factors of ≈7.6 and ≈2.2 compared to our present 1800 nm MDPs, potentially approaching the performance limit of extended films.

Beyond these particle‐level design strategies, our findings also highlight the broader implications of SF MDPs for imaging technology. Their superior performance compared to conventional SP tracers enables not only higher spatial resolution, but also a significant reduction in the magnetic field gradients required for MPI. This opens the door to simpler, more compact, and energy‐efficient scanner designs, paving the way toward clinically viable and widely deployable systems.

The top‐down fabrication approach based on sputter‐deposited multilayers further offers unmatched flexibility in materials selection and control over particle geometry and architecture. Future work should focus on optimizing these parameters to mitigate demagnetization effects and maximize tracer efficiency. Such advances will be key to fully unlocking the transformative potential of SF MDPs in next‐generation biomedical imaging, with particular promise for vascular imaging applications where smaller tracer dimensions are essential.

Although our proof‐of‐concept flow is not yet optimized for throughput, wafer‐scale sputtering and nanoimprint or interference lithography, combined with lift‐off or parallel plasma etching, offer clear industrial scale‐up routes.

In short, careful engineering of DMIM layer geometry opens a route to biologically compatible MPI tracers without loss of performance.

## 4 Experimental Section

### Silicon Wafers

As a substrate, we used 2‐inch Prime Si wafers with a 100nm thermal ${\mathrm{SiO}}_{2}$ layer (1‐side polished, p‐type (Boron), ρ = 1.10 Ωcm, thickness = (279 ± 20) μm purchased from MicroChemicals GmbH.

### Multilayer Stack

Ti, Ge, ${\mathrm{Co}}_{40}{\mathrm{Fe}}_{60}$, and ${\mathrm{Al}}_{2}O_{3}$ sputtering targets, all with a purity of 99.99 %, from HMW Hauner were used for the sputter deposition of the layers.

### Silicon Nitride TEM Windows

For transmission electron microscopy (TEM) studies, we used ${\mathrm{Si}}_{3}N_{4}$ TEM window grids with a 5nm thick silicon nitride membrane, purchased from Electron Microscopy Sciences (Cat. #76042‐44, Lot #210503).

### Polysterene Bead Mask

Polystyrene Carboxylate‐Modified Nanospheres were purchased at microParticles GmbH, Berlin, Germany. The particles sizes and standard‐deviations of (5310 ± 100), (2230 ± 40), and (658 ± 15) nm, respectively, and were purchased in suspensions with a solid content of 10% (5310 and 2230 nm beads) and 5% (658 nm beads)

### Perimag

The dextran‐coated iron‐oxide nanoparticles (Perimag, *c*_Fe_ = 8.5 mg ml^−1^, *d*_hydr_ = 130nm) used for the comparison were purchased from Micromod Partikeltechnologie GmbH, Rostock, Germany.

### Fabrication of DMIM MDP

DMIM MDPs were fabricated by sputter‐deposition and microfabrication using 2‐inch Si wafers as substrates. The multilayers, including the sacrificial 50 nm‐thick Ge layers were grown on the Si wafer using a AJA DC/RF magnetron sputtering system with a working pressure of ≈3.5μbar Ar and a base pressure of ≈1 × 10^−8^mbar. The fabrication steps following the multilayer deposition onto the Si Substrates are illustrated in Figure 3a–d.

Nominal layer thicknesses (in nanometers) were obtained by sputtering at a predefined power and deposition time. The sputtering parameters were chosen such that the intended thickness was reached over several tens of seconds, i.e., much longer than the shutter open/close time of the sputter gun. Thickness calibration was performed using X‐ray reflectometry (XRR) on thicker test layers (typically several tens of nanometers) giving layer thicknesses with sub‐0.1 nm precision.

On top of the uppermost Ge layer a hard mask out of carboxylate‐modified polysterene (PS) beads were self‐assembled at the water–air interface. This method created a highly ordered hexagonal packed monolayer of PS beads, which was deposited on top of the multilayer stack by slow water evaporation.^[^ ^29^, ^35^ ^]^ In a next step, the PS beads were treated with oxygen plasma for 8.5, 16, and 19 min with a power of 40 W to separate them out and to reach a reduced diameter of (530 ± 30), (1800 ± 200), and (4800 ± 400) nm. To cut the disk out of the multi layer stack an Ar ion‐miller from Oxford Instrument was used with a working pressure of ≈3.5 μbar. The milling was performed at 600 V with 300 mA at an angle of 90° to the sample surface. The Ge top and bottom sacrificial layers were then dissolved in a final step during 1.5 h in 35wt% $H_{2}O_{2}$ to release the MDP. Although no additional surface functionalization was applied in this proof‐of‐concept study, the sputter‐deposition approach readily allows the addition of capping layers that can be tailored for subsequent wet‐chemical functionalization to improve colloidal stability and biocompatibility.^[^ ^36^ ^]^

### Scanning Electron Microscopy (SEM)

SEM was performed on the DMIM MDPs immobilized on the wafer and following detachment using a Hitachi SU5000 with a tilt stage. Particle size distributions were analyzed based on SEM micrographs (>100 particles were manually counted per sample).

### Scanning Transmission Electron Microscopy (STEM)

For the scanning transmission electron microscopy (STEM) assessments, Ti(5)/Al_2_O_3_(3)/CoFe(*t*_CoFe_)/Al_2_O_3_(3) trilayers were sputter‐deposited on Si_3_N_4_ windows with a thickness of 5 nm. These samples were imaged on a Talos F200X electron microscope (Thermo fisher scientific) in STEM mode using a high angle annular darkfield (HAADF) detector.

### Vibrating Sample Magnetometry Measurements

The *M*(*H*) data presented here were measured with a 8600 Series VSM by “Lakeshore Cryotronics, Inc” with a maximum field of 3.62 T. Measurements were conducted at room temperature. Thin film and MDP samples were measured in‐plane. For background correction, the slope of the linear part of the measured *M*(*H*) curves at the high field range $\mu_{0}H_{a}\geq 500\;\mathrm{mT}$ was subtracted. Note that VSM measures the magnetic moment (A m^2^), from which the magnetization in A m^−1^ was calculated by dividing by the sample volume. For the extended DMIM and the DMIM MDPs, the total disk areas and the total nominal CoFe thickness were used, whereas for Perimag the Fe_3_O_4_ volume content was considered.

### 1D Magnetic Particle Spectroscopy

All presented 1D MPS measurements were conducted in a custom built spectrometer^[^ ^30^ ^]^ at a drive field frequency of 25 kHz with an amplitude of 20mT. All measurements were averaged for 0.2 s and background and transfer function (TF) corrected.^[^ ^37^ ^]^ For the x‐space PSF a set of 1D MPS measurements were conducted with superimposed offsets from –25 to 25 mT. Then, the TF‐corrected voltage at the time of the maximum positive slope of the excitation field is extracted for each offset and visualized as the PSF. Due to imperfect background correction the fundamental component had to be recovered.

### Inductively Coupled Plasma Mass Spectrometry (ICPMS)

The magnetic mass concentrations of the MDP suspension were determined through inductively coupled plasma mass spectrometry (ICP‐MS). To this end, 50μ l of the respective stock sample were digested in quartz tubes using a mixture of 0.75 ml hydrochloric acid (37 pc, Normatom, VWR) and 0.25 ml nitric acid (69 pc, Normatom, VWR) in a pressurized microwave (TurboWAVE, MLS GmbH, Germany) at 230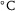 and 120 bar for 19 min. Subsequently, the samples were transferred into 50 ml Falcon tubes and filled up with ultrapure water to the mark. All samples were then analyzed for the defining magnetic elements Fe and Co using a 7900 single‐quadrupole ICP‐MS (Agilent Technologies, CA). The elements were quantified using the isotopes ^56^Fe and ^59^Co. Non‐spectral interferences were corrected using an internal standard, containing ^103^Rh, which was mixed online with the sample. Calibration was performed with certified element standards (Inorganic Ventures, Christiansburg, VA, USA) diluted in the same acid matrix as the samples (0.75 ml HCl/0.25 ml ${\mathrm{HNO}}_{3}$ in 50 ml ultrapure water).

### 1D Magnetic Particle Spectrum Normalization

To ensure comparability, the higher harmonic amplitudes of the 1D spectra shown in Figure 3h and Figure S2b (Supporting Information) were given in arbitrary units per magnetic mass (*m*_Fe_ for Perimag and *m*_CoFe_ for the DMIMs and MDP). The MDP tracers with diameters of 1800 and 530 nm, as well as Perimag, were measured in 20μl of suspensions with identical magnetic mass concentrations of $c_{\text{CoFe}}^{\text{MDP}}=c_{\text{Fe}}^{\text{Perimag}}=$272μg ml^−1^. The total magnetic mass in the respective samples was thus given by 5.44 μg. The magnetic mass concentrations of the 500  and 1800 nm MDP stock suspensions were determined via ICPMS and diluted with deionized water, if desired. The unpatterned DMIMs covered a wafer with the area of $\approx 4.1\;{\mathrm{mm}}^{2}$, leading to a total magnetic mass of around 337 ng. The measured harmonic amplitudes were thus scaled by a factor of ≈16. The concentration of the 4600 nm MDP suspension was not quantified in this experiment. To enable a comparative analysis of the spectral characteristics, its signal spectrum was normalized to amplitude of the 3rd harmonic of the 1800 nm MDP suspension. This normalization results in a conservative estimate of the signal amplitudes for the 4600 nm diameter MDPs.

### Magnetic Particle Imaging (MPI)

The MPI imaging experiments were conducted in the Bruker preclinical MPI system (Bruker MPI 20/25 FF, Bruker BioSpin GmbH & Co. KG, Ettlingen, Germany). As the excitation field a 2D Lissajous trajectory with *f*_*x*_ = 2.5 MHz/102 and *f*_*y*_ = 2.5 MHz/96 with 12 mT/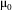 was used. The system matrix (SM) was determined by measuring the signal from a cubic 8μL sample that was sequentially positioned at different locations within the selection field. The magnetic field gradient was set to 0.66, 0.66, and 1.33  T m^−1^ along the *x*, *y*, and *z* axes, respectively. In total 36 × 36 positions using 1 mm steps were acquired covering the drive field FOV without overscan in the *xy* plane. Each of the eight phantoms was imaged for around 1 min with 2.1 s of averaging per frame, only the last frame is shown and was used for the evaluation.

### Hybrid MPI

Similar to the trajectory in the Bruker scanner a 2D Lissajous trajectory with 12 mT was used in the custom‐built magnetic particle spectrometer^[^ ^34^ ^]^ with excitation frequencies *f*_*x*_ = 2.5 MHz/102 and *f*_*y*_ = 2.5 MHz/99. For the hybrid reconstruction two system matrices with different grids were measured for each tracer to avoid inverse crime.^[^ ^38^ ^]^ The system matrix, which was used for the generation of the emulated measurement signal had a size of 101 × 101 positions (0.3 mT steps), while the SM used for reconstruction had a size of 61 × 61 (0.5 mT steps). Note that in contrast to the SM acquired in the MPI system these offsets overscan the drive field FOV by 25%. Due to the larger employed sample volume and the higher SNR provided by the magnetic particle spectrometer, compared to the MPI measurements, the measurements were only averaged for 0.21 s.

The concentration distribution of the hybrid phantoms presented in Figure 4e is given by

(1) $$c\left(x,y,\theta \right)=\frac{1}{2}\cos\left(2\pi \;\;\frac{x\cdot \cos\theta +y\cdot \sin\theta }{\lambda }\right)+\frac{1}{2}$$

and restricted to a circular ROI with a radius of 10 mT. Outside this ROI, the concentration is zero. The modulation transfer functions is calculated using seven different orientations of the stripe pattern (θ ∈ {0°, 15°, 30°, 45°, 60°, 75°, 90°}) for each wavelength. The wavelength λ can be converted from the offset domain to a spatial dimension using a gradient strength *G* as λ_*G*_ = λ/*G*.

The coronary artery phantoms (Figure 4f) were created by calculating projection images using segmented 3D CT images from the open ImageCAS dataset.^[^ ^39^ ^]^ The stenoses were added manually to the projection images.

### MPI Image Reconstruction

All MPI images, both from the scanner as well as the hybrid measurements, were reconstructed using the L2‐regularized Kaczmarz method implemented in the open‐source software framework MPIReco.jl.^[^ ^40^ ^]^ First, all frequencies were inversely weighted by their noise level estimated from the background of the system matrix measurement, resulting in a noise‐whitened spectrum, which is beneficial for a robust reconstruction. After that all frequency components above the fundamental frequency with a signal‐to‐noise ratio above two were used for the reconstruction with 10 iterations while constraining the solution to real positive values. For the reconstructions done with the Bruker system the same normalized regularization parameter λ = 10^−3^ was used for both particles. Due to the higher SNR in the hybrid reconstruction it was possible to use a smaller value; however, due to differences in the visible noisiness of the images the regularization parameter was selected for each tracer individually (λ_MDP_ = 10^−5^, λ_Perimag_ = 5 · 10^−5^), resulting in comparable noise in the image. A comparison for different regularization parameters is given in the Supporting Information.

## Conflict of Interest

E.M., I.K.H., and H.J.H. declare coinventorship on a patent application (EP 24163001.1) filed by Empa and ETH Zurich. All other authors declare no conflict of interest.

## Author Contributions

H.J.H. and I.K.H. conceived the project. E.M. deposited the magnetic multilayer films, and performed the magnetometry experiments and data analysis. The polystyrene sphere self‐assembly and successive oxygen plasma etching to reduce the PS sphere diameters was performed by M.K., while E.M. performed the successive ion etching to pattern the MDPs and successively release these from the wafer support to form the MDP suspensions with experimental support from S.S. A.G. performed the chemical analysis to determine the concentration of MDP suspensions. The MPS and MPI data acquisition and processing was performed by E.M. and J.A., under the supervision of M.G. The manuscript was conceived by E.M., J.A., M.G., H.J.H., and I.K.H., and all authors discussed and contributed to the final manuscript.

## Supporting information

Supporting Information

## Data Availability

The data that support the findings of this study are available from the corresponding author upon reasonable request.
