# Supplementary material for: Superferromagnetic Disk Particles for Magnetic Particle Imaging
Source: Small Methods. 2025 Nov 23;9(12):e01349. doi: 10.1002/smtd.202501349 (PMC12716192; doi:10.1002/smtd.202501349)
Supplement: Supplementary file 1 — Supporting Information [file SMTD-9-e01349-s001.pdf]

Supporting Information; Superferromagnetic Disk Particles for Magnetic Particle Imaging

E. M. Mayr,[1,2,3,4,5] J. Ackers,[6] A. Gogos,[4] S. Scheibler,[1,5,4]
M. Krupiński,[7] M. Graeser,[6,8] I. K. Herrmann,[1,2,3,4,*] and H. J. Hug[5,9,*]

[1]*Nanoparticle Systems Engineering Laboratory, Department of Mechanical and Process Engineering, ETH Zurich, Sonneggstrasse 3, 8092 Zurich, Switzerland*
[2]*Faculty of Medicine, University of Zurich, Raemistrasse 71, 8006 Zurich, Switzerland*
[3]*Ingenuity Lab, Balgrist University Hospital and University of Zurich, Forchstrasse 340, 8008 Zürich, Switzerland*
[4]*Nanomaterials in Health Laboratory, Swiss Federal Laboratories for Materials Science and Technology (Empa), Lerchenfeldstrasse 5, 9014 St. Gallen, Switzerland.*
[5]*Magnetic & Functional Thin Films Laboratory, Swiss Federal Laboratories for Materials Science and Technology Empa, Ueberlandstrasse 129, 8600 Dübendorf, Switzerland*
[6]*Fraunhofer IMTE, Fraunhofer Research Institution for Individualized and Cell-Based Medical Engineering, Mönkhofer Weg 239a, 23562 Lübeck, Germany*
[7]*Institute of Nuclear Physics, Polish Academy of Sciences, Radzikowskiego 152, 31-342 Kraków, Poland*
[8]*Chair of Metrology, University Rostock, Albert-Einstein-Str. 2, 18059 Rostock, Germany*
[9]*Department of Physics, University of Basel, Klingelbergstrasse 82, 4056 Basel, Switzerland*

SUPPLEMENTARY INFORMATION

Dead Magnetic Layer

At interfaces between magnetic and non-magnetic materials, magnetic properties can be altered significantly. In particular, magnetic strata with reduced magnetization, often referred to as "dead layers", can form near the interfaces, leading to a reduced overall saturation magnetization in thinner films [1–3] (Fig. S1**a**). This behavior is commonly modeled by assuming a central magnetic layer with bulk-like, thickness-independent properties, flanked by interfacial regions with suppressed magnetization. To quantify these effects, we fabricated a series of samples with nominal magnetic layer thicknesses ranging from 0.8 to 15 nm. The magnetic moment per unit sample area, measured by vibrating sample magnetometry, is shown in Fig. S1**b**. The data reveal the expected linear dependence of the areal magnetic moment on the nominal film thickness. The linear fit yields a saturation magnetization of $M_s = (1.92 \pm 0.01)\,\mathrm{MA\,m^{-1}}$, which agrees well with the literature value $M_s = 1.95\,\mathrm{MA\,m^{-1}}$ reported for $Co_{45}Fe_{65}$ [4]. The x-axis intercept at $t_{dml} = (0.56 \pm 0.04)\,\mathrm{nm}$, corresponds to the combined thickness of the top and bottom dead magnetic layers. Notably, data points for films thinner than 1.4 nm fall slightly below the fitted line. We attribute this additional reduction in magnetic moment to an increased density of non-exchange-coupled superparamagnetic islands that form in ultrathin superferromagnetic films, further diminishing the net magnetization at room temperature. We note that, due to the UHV sputter deposition from metallic CoFe and Al_3O_3 targets, the layers are fully metallic apart from interfacial oxidation at the CoFe/Al_3O_3-interfaces. The resulting dead layer thickness and saturation magnetization are quantitatively determined by VSM (Fig. S1**b**). Since sputter-deposited CoFe and Al_3O_3 are polycrystalline with no preferential orientation, further XRD or XPS characterization would not provide addi-tional information relevant to tracer optimization.

* Corresponding authors: hans-josef.hug@empa.ch and ingeh@ethz.ch

Influence of Particle Diameter on Magnetic Properties and Signal Generation

While the enhanced MPS signal and improved MPI performance of the 1800 nm-diameter MDPs relative to Perimag are highlighted in the main manuscript, the even stronger response observed in extended DMIM films underscores the untapped potential of our approach. To better understand this trend, Fig. S2**a** and **b** compare the VSM $M(H)$ loops and MPS spectra obtained for an extended DMIM film, MDPs with diameters of 4600, 1800, and 530 nm and Perimag. The measured magnetic susceptibilities increase with particle size, from $\chi_{\mathrm{VSM}}^{\mathrm{MDP}} = 58$ to 139 and 866 for 530 nm, 1800 nm and 4600 nm disks, respectively. For the extended film, which exhibits a hysteresis loop, we determine the local susceptibility at the coercive field as $\chi|_{H\mathrm{c}} := \frac{\mathrm{d}M}{\mathrm{d}H}|_{H\mathrm{c}} = 18'300$. Notably, the smallest MDPs (530 nm) exhibit a susceptibility lower than that of Perimag ($\chi_{\mathrm{VSM}}^{\mathrm{Perimag}} \approx 123$), whereas larger disks exhibit significantly higher values.

A corresponding trend is observed in the MPS signal amplitudes, which increase with particle size. While the 530 nm disks produce weaker higher harmonic signals than Perimag (teal and purple curves in Fig. S2**b**), both the 1800 nm and 4600 nm MDPs show markedly improved signal strengths across a wide harmonic range. This behavior is consistent with the size-dependent increase in local magnetic susceptibility, as evident from the steepening slopes in the VSM $M(H)$ loops. These results collectively highlight the promising potential of optimizing MDP geometry to further enhance MPI tracer performance.

The observed changes in magnetic behavior with decreasing disk diameter, and the associated reduction in MPI signal generation for smaller MDPs, can be attributed to increasing demagnetization effects. These arise from the internal demagnetizing field H_d, which opposes the net magnetization and causes a progressive tilting of the $M(H)$ loops, as clearly

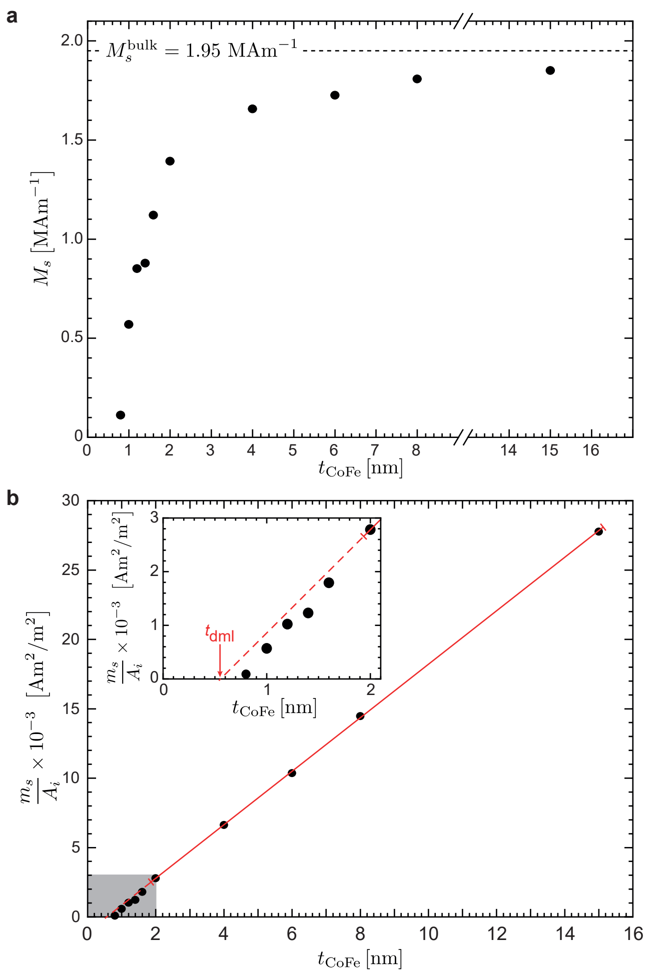

FIG. S1. **a** Saturation Magnetization obtained for $[\mathrm{CoFe/Al_2O_3}]_{\times n}$ $/\mathrm{Al_2O_3}$ multilayers with the nominal CoFe layer thickness $t_{\rm CoFe}$ ranging from 0.8 nm to 15 nm. The number of magnetic layers is given by $n = 1$ for the $t_{\rm CoFe} = 15$ nm and $n = 4$ for the other ones. **b** Saturation moment per metal/oxide interface area $A_{\rm i}$. The solid line shows a linear fit ($t_{\rm CoFe} \geq 2$ nm), the dashed line shows its interpolation. The inset shows a zoomed-in section, indicated by the shaded gray area.

seen in the VSM measurements (Fig. S2**g**). For a homogeneously magnetized spheroid, the demagnetizing field is well described by the geometric demagnetizing factor N via the relation $H_d = -N \cdot M$ [4]. In contrast, a cylindrical disk with in-plane magnetization exhibits a highly inhomogeneous internal field. Nevertheless, an approximate expression for an effective demagnetizing factor—assuming a spatially uniform H_d—has been derived by Sato et al. [6],

$$N_{\rm disk} = \left(2 + \frac{\sqrt{\pi}D}{2t}\right)^{-1}, \qquad \text{(S1)}$$

where D is the disk diameter and t the disk thickness. Notably, $N \to 0$ for large D/t ratios, and $N \to \frac{1}{2}$ as D/t becomes small.

The strong intra-layer exchange coupling in the DMIM structure, which gives rise to its superferromagnetic (SF) be-

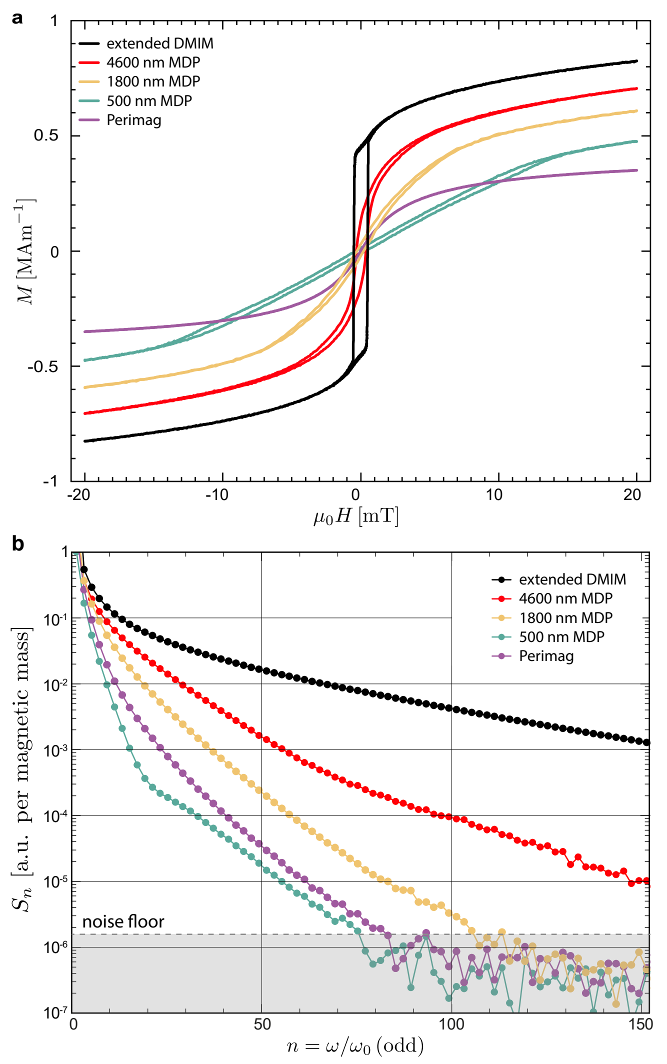

FIG. S2. **a** $M(H)$-loops measured over a field range of ± 20 mT for the extended DMIM film (black line), and for disk particles with diameters of 530, 1800 and 4600 nm (teal, yellow and red lines), compared to Perimag (purple). The magnetization was calculated by dividing the measured magnetic moment by the volume of the magnetic material. **b** Odd higher harmonic amplitudes, normalized to magnetic mass, measured by magnetic particle spectroscopy [5] using a 25 kHz oscillatory field with an amplitude of $\mu_0 H = 20$ mT, for the extended SF DMIM film (black), as well as suspensions of 530, 1800 and 4600 nm diameter disk particles (teal, yellow and red lines) and Perimag (purple).

havior, ensures that the islands in each magnetic disk particle (MDP) acts as a single magnetic entity. This effective coupling links the CoFe islands across multiple layers, resulting in a three-dimensionally connected magnetic network embedded in an $\mathrm{Al_2O_3}$ matrix. As a result, despite their multilayered composition, the MDPs can be reasonably approximated as homogeneously magnetized cylindrical disks for the purpose of modeling demagnetization effects. The effective thickness $t_{\rm eff}$ relevant for estimating the demagnetization factor there-

fore includes both the magnetic (CoFe) and intervening non-magnetic (Al_2O_3) layers. For ten CoFe layers separated by nine Al_2O_3 layers, this gives $t_{\mathrm{eff}} = 10 \cdot t_{\mathrm{CoFe}} + 9 \cdot t_{\mathrm{Al_2O_3}} = 16.75\,\mathrm{nm}$. Inserting this value into Equation S1 yields estimated demagnetization factors of $N \approx 0.03$, 0.01, and 0.004 for disk diameters of 530, 1800, and 4600 nm, respectively.

Assuming that the patterning process does not alter the intrinsic magnetic properties of the DMIMs, the relationship between the intrinsic susceptibility of the DMIMs χ_{i} and the externally measured, geometry dependent susceptibility for a disk, $\chi'_{\mathrm{geom.}}$, is given by [4]

$$\chi'_{\mathrm{geom.}} = \left(\frac{1}{\chi_{\mathrm{i}}} + N_{\mathrm{disk}}\right)^{-1}. \qquad \text{(S2)}$$

Note that unpatterned DMIMs with lateral dimensions on the millimeter scale are virtually unaffected by H_d. The internal susceptibility of the multilayers thus equals the susceptibility measured for the extended DMIMs $\chi_{\mathrm{i}} = \chi_{\mathrm{DMIM}}^{\mathrm{VSM}} \approx 18'300$.

The susceptibilities $\chi'_{\mathrm{geom.}}$ obtained from Equation S2 approximate those extracted from $M(H)$-loops where the magnetization is calculated by distributing the measured magnetic moments over the effective thickness of the DMIM structure, $t_{\mathrm{eff}} = 10 \cdot t_{\mathrm{CoFe}} + 9 \cdot t_{\mathrm{i}} = 16.75\,\mathrm{nm}$. However, for the $M(H)$-loops plotted in Fig. 3**g** (main text) and Fig. S2**a**, the magnetization was calculated by attributing the magnetic moment solely to the CoFe layers, and, in the case of Perimag, to the volume content of the magnetic material. Therefore, to enable a direct comparison, the susceptibilities calculated from Equation S2 must be rescaled by a factor of $t_{\mathrm{eff}}/(10 \cdot t_{\mathrm{CoFe}}) = 1.675$ to account for the different normalization of the magnetization. The rescaled calculated susceptibilities $\chi_{\mathrm{geom.}}$ are compared to the experimental susceptibilities χ_{VSM} extracted from the measured $M(H)$-loops depicted in Fig. S2**a**. The results are listed in table Table S1.

D	530 nm	1800 nm	4600 nm
χ_{VSM}	58	139	866
$\chi_{\mathrm{geom.}}$	50	163	411
$\chi_{\mathrm{geom.}}/\chi_{\chi_{\mathrm{VSM}}}$	0.86	1.16	0.45

TABLE S1. Overview over the susceptibilities observed in VSM measurements and obtained from the demagnetization model, taking into account the geometry of the disks with an effective magnetic layer thickness of $t_{\mathrm{eff}} = 16.75\,\mathrm{nm}$ and respective diameters of 530, 1800, and 4600 nm.

The experimentally observed susceptibilities of the 530 and 1800 nm diameter MDPs are reasonably well described by the values obtained from our demagnetization model, whereas the susceptibility of the 4600 nm MDPs is underestimated by more than a factor of two. This discrepancy can be understood by recognizing that the description of the demagnetizing field through a demagnetizing factor can only approximate the inhomogeneous field inside the MDP. In disks with a large diameter-to-thickness ratio, the demagnetization field is strongest near the disk edges and significantly weaker in the interior, such that the approximation used in Equation S1 overestimates the effective demagnetization effect. Nevertheless, the consideration of demagnetization effects with this simple model explains the gradual loss of MPI signal observed for smaller-diameter MDPs.

Hybrid MPI reconstruction parameters

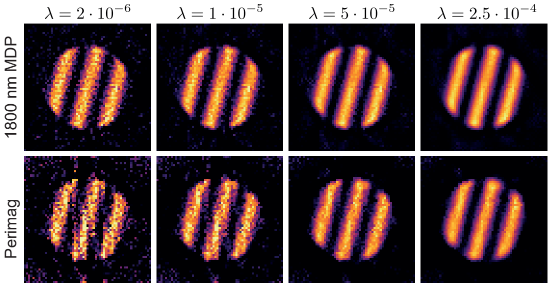

FIG. S3. Comparison of different regularization parameters in the hybrid reconstruction for both the 1800 nm MDPs and Perimag. Due to the properties of the inverse problem, the same regularization parameter gives perceptually more noisy results for the Perimag reconstruction. For the main evaluation, reconstruction parameters of $\lambda = 10^{-5}$ and $\lambda = 5 \cdot 10^{-5}$. have been used for the MDPs and Perimag respectively.

In system matrix based reconstruction for MPI the resolution and signal-to-noise ratio are strongly intertwined [7, 8]. Depending on the signal-to-noise ratio of the measurement, different regularization parameters shift the balance between image sharpness and smoothness, leading to either noisy images with potentially higher resolution or smoother images with reduced feature separation. For the investigation in the main part utilizing the hybrid reconstructions a visually equivalent result regarding the noisiness of the reconstruction result was chosen using reconstructions of sine patterns which were not close to the resolution limit. Fig. S3 shows multiple reconstructions of such a sine pattern with a 6.66 mT period, using different regularization parameters for both tracers. In the direct comparison at a fixed λ the Perimag reconstruction appears significantly noisier, with visual equivalence roughly reached at a ratio of $\frac{\lambda_{\mathrm{Perimag}}}{\lambda_{\mathrm{MDP}}} = 5$. To get the best possible resolution and images which are not overly smoothed by the regularization, the regularization parameters were chosen as $\lambda_{\mathrm{MDP}} = 10^{-5}$ and $\lambda_{\mathrm{Perimag}} = 5 \cdot 10^{-5}$.

System matrix analysis

Fig. S4 shows additional components of the 2D system matrices, complementing the data presented in Fig. 4**d** of the main manuscript. The previous figure showed a grayscale representation to directly compare the signal amplitudes of MDP and Perimag tracers with the same intensity range. Fig. S4 on the other hand uses a color scale that shows both phase and amplitude at once. Specifically, the signal phase angle is represented by the hue, while the signal amplitude is conveyed

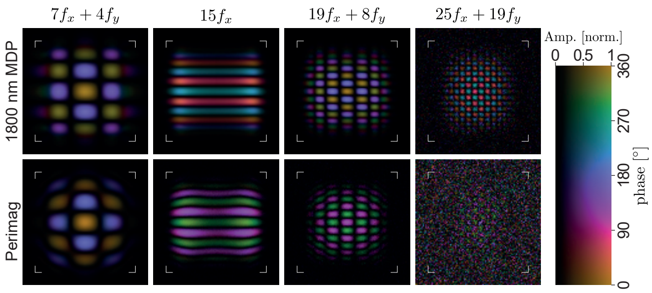

FIG. S4. System matrix components of 1800 nm MDPs and Perimag under 2D excitation in complex colorscale, encoding amplitude and phase. The SM structure of the MDPs is closer to the ideal Chebychev polynomials and higher spatial frequency components are visible above the noise floor.

through color brightness. To highlight structural differences between individual matrix components, the brightness is normalized separately for each panel. As a result, the present visualization emphasizes the spatial and phase characteristics of each component, rather than enabling direct amplitude comparisons across tracers.

The system matrix components of the MDP tracers exhibit a highly regular checkerboard-like pattern, reminiscent of Chebyshev polynomials that mathematically describe the response of tracer particles with ideal step-like magnetic switching under Lissajous excitation [9]. The MDP patterns remain well-defined and spatially distinct across the whole excitation field of view (FOV), indicated by white markers. In contrast, the Perimag tracer shows a tendency toward peak merging and reduced spatial separation, particularly near the outer edges of the FOV, indicating a loss of resolution in those regions. In addition to the pattern changes also the amplitude drop-off towards larger offsets differs for Perimag and the MDPs, especially visible in the third column of Fig. S4. While for Perimag the amplitude of the signal decays significantly towards the edge of the excitation FOV, more peaks of the MDP patterns are visible at the chosen colorscale. An additional, currently unexplained phenomenon is a slight phase shift observed in the MDP signals near the edges of the FOV, visualized by subtle hue variations in the corners. Rather than undergoing a sharp transition from positive to negative phase through a zero-crossing, the components exhibit a more gradual phase shift without a clear zero point. This spatial phase variation may enhance image encoding, as it introduces richer phase information into the system matrix compared to cases where each component maintains a single, uniform phase. Such phase diversity could contribute to improved reconstruction accuracy.

Beyond improved encoding performance at the same harmonics, the enhanced high-frequency signal generation of the MDPs enables the capture of additional harmonic components above the noise floor (see fourth column in Fig. S4), thereby extending the range of spatial frequencies that can be encoded and improving resolution, contributing to better imaging performance.

[1] K. Oguz, P. Jivrajka, M. Venkatesan, G. Feng, and J. M.D. Coey. Magnetic dead layers in sputtered co40 fe40 b20 films. Journal of Applied Physics, 103, 2008.

[2] J. Li, Z. Y. Wang, A. Tan, P. A. Glans, E. Arenholz, C. Hwang, J. Shi, and Z. Q. Qiu. Magnetic dead layer at the interface between a co film and the topological insulator bi 2se 3. Physical Review B - Condensed Matter and Materials Physics, 86, 8 2012.

[3] S. B. Porter, M. Venkatesan, P. Dunne, B. Doudin, K. Rode, and J. M.D. Coey. Magnetic dead layers in la0.7sr0.3mno3 revisited. IEEE Transactions on Magnetics, 53, 11 2017.

[4] J. M. D. Coey. Magnetism and Magnetic Materials. Cambridge University Press, 2010.

[5] M. Graeser, A. Von Gladiss, M. Weber, and T. M. Buzug. Two dimensional magnetic particle spectrometry. Physics in Medicine and Biology, 62:3378–3391, 4 2017.

[6] M. Sato and Y. Ishii. Simple and approximate expressions of demagnetizing factors of uniformly magnetized rectangular rod and cylinder. Journal of Applied Physics, 66:983–985, 1989.

[7] J. Weizenecker, J. Borgert, and B. Gleich. A simulation study on the resolution and sensitivity of magnetic particle imaging. Physics in Medicine and Biology, 52:6363–6374, 11 2007.

[8] Tobias Knopp, Sven Biederer, Timo F Sattel, Marlitt Erbe, and Thorsten M Buzug. Prediction of the spatial resolution of magnetic particle imaging using the modulation transfer function of the imaging process. IEEE transactions on medical imaging, 30(6):1284–1292, 2011.

[9] Jürgen Rahmer, Jürgen Weizenecker, Bernhard Gleich, and Jörn Borgert. Signal encoding in magnetic particle imaging: Properties of the system function. BMC Medical Imaging, 9, 4 2009.
